# Cardiovascular, Neurological, and Immunological Adverse Events and the 23-Valent Pneumococcal Polysaccharide Vaccine

**DOI:** 10.1001/jamanetworkopen.2023.52597

**Published:** 2024-01-22

**Authors:** Dongwon Yoon, Ha-Lim Jeon, Ju Hwan Kim, Hyesung Lee, Ju-Young Shin

**Affiliations:** 1 School of Pharmacy, Sungkyunkwan University, South Korea; 2 Department of Biohealth Regulatory Science, School of Pharmacy, Sungkyunkwan University, South Korea; 3 School of Pharmacy and Institute of New Drug Development, Jeonbuk National University, South Korea; 4 Samsung Advanced Institute for Health Sciences and Technology, Sungkyunkwan University, South Korea

## Abstract

**Importance:**

Despite widespread immunization with the 23-valent pneumococcal polysaccharide vaccine (PPSV23), safety concerns remain owing to a lack of statistical power and largely outdated evidence.

**Objective:**

To evaluate the association between cardiovascular, neurological, and immunological adverse events and PPSV23 vaccination in older adults.

**Design, Setting, and Participants:**

This population-based cohort study using a self-controlled risk interval design used a large linked database created by linking the Korea Immunization Registry Information System and the National Health Information Database (2018 to 2021). Participants included patients aged 65 years or older with a history of PPSV23 vaccination and incident cardiovascular, neurological, or immunological events during the risk and control intervals. Data were analyzed from November 2022 to April 2023.

**Exposure:**

23-valent pneumococcal polysaccharide vaccine.

**Main Outcomes and Measures:**

The occurrence of 1 among 6 cardiovascular events (myocardial infarction, atrial fibrillation, cardiomyopathy, heart failure, hypotension, and myocarditis or pericarditis), 2 neurological events (Bell palsy and Guillain-Barré syndrome), and 3 immunological events (sepsis, thrombocytopenia, and anaphylaxis) during the risk and control periods. The risk and control intervals were defined as 1 to 28 and 57 to 112 days after PPSV23 vaccination, respectively. Conditional Poisson regression was used to estimate the incidence rate ratio (IRR) with a 95% CI.

**Results:**

Altogether, 4355 of the 1 802 739 individuals who received PPSV23 vaccination and experienced at least 1 outcome event were included (mean [SD] age, 72.4 [8.2] years; 2272 male participants [52.1%]). For cardiovascular events, there were no significant associations for myocardial infarction (IRR, 0.96; 95% CI, 0.81-1.15), heart failure (IRR, 0.85; 95% CI, 0.70-1.04), and stroke (IRR, 0.92; 95% CI, 0.84-1.02). Similarly, no increased risks were observed for neurological and immunological outcomes: Bell palsy (IRR, 0.95; 95% CI, 0.72-1.26), Guillain-Barré syndrome (IRR, 0.27; 95% CI, 0.06-1.17), sepsis (IRR, 0.99; 95% CI, 0.74-1.32), and thrombocytopenia (IRR, 1.18; 95% CI, 0.60-2.35).

**Conclusions and Relevance:**

In this self-controlled risk interval study, there was no appreciable increase in risk for most cardiovascular, neurological, or immunological adverse events following PPSV23. The updated safety profile of PPSV23 provides supportive evidence for the establishment of immunization strategies for older adults.

## Key Points

**Question:**

Does vaccination with 23-valent pneumococcal polysaccharide vaccine (PPSV23) increase the risk of cardiovascular, neurological, and immunological adverse events in elderly adults?

**Findings:**

In this cohort study that included 4355 older adults who received PPSV23 vaccination and experienced cardiovascular, neurological, or immunological events, the risk of these events did not increase during the prespecified risk interval (1-28 days after PPSV23 vaccination) compared with the control interval (57-112 days after PPSV23 vaccination).

**Meaning:**

This updated safety evidence for PPSV23 suggests support for previous findings that PPSV23 is not associated with a higher risk of serious systemic adverse events of interest.

## Teaser

This cohort study evaluates the association between cardiovascular, neurological, and immunological adverse events and the 23-valent pneumococcal polysaccharide vaccine in older adults.

## Introduction

Globally, pneumonia remains one of the leading causes of mortality in older adults, resulting in approximately 2.5 million deaths annually.^1,2^ Consequently, intensive management strategies are becoming increasingly important for public health, which has lead to increased immunization policies for prevention of pneumonia. According to the current recommendations of the Advisory Committee on Immunization Practices, all older adults should receive 1 dose of the 23-valent pneumococcal polysaccharide vaccine (PPSV23).^3^ Consistent with these recommendations, the Korea Disease Control and Prevention Agency implemented a single-dose schedule of the PPSV23 vaccine in its national immunization program beginning in May 2013 for adults aged 65 years or older.^4^ To date, over 5 million older adults have received PPSV23, and the vaccination rate is reported to be 56.2%.^5^

PPSV23 is generally deemed safe based on more than 30 years of evolving clinical experience.^6^ Most adverse events following PPSV23 vaccination are mild and transient in nature and resolve within a few days.^7^ Previous studies have reported no evidence of an increased risk of cardiovascular outcomes in older adults following PPSV23 vaccination.^8,9^ Notably, a meta-analysis suggested potential cardioprotective effects of the pneumococcal vaccine, including reduced risk of myocardial infarction and all-cause mortality.^10^ However, these studies were generally limited by statistical power or residual confounding, and the current knowledge is predominantly based on White populations. Moreover, recent pharmacovigilance studies have identified potential signals of PPSV23 for various outcomes,^11,12,13^ necessitating further signal evaluation studies.

Given the importance of obtaining updated evidence and massive immunization with PPSV among the elderly population in South Korea, it is crucial to conduct a study that minimizes the proposed limitations by using a self-controlled design to provide public confidence regarding the safety of PPSV23. Therefore, our objective was to assess the potential association between cardiovascular, neurological, and immunological adverse events and PPSV23 vaccination in older adults using a nationwide large linked database.

## Methods

This study was approved by the institutional review board of Sungkyunkwan University, which waived the requirement for informed consent because only deidentified data were used. This study followed the Strengthening the Reporting of Observational Studies in Epidemiology (STROBE) reporting guideline.

### Data Sources

We assembled a large linked database by linking the Korea Immunization Registry Information System (KIRIS) to the National Health Information Database (NHID) from July 2018 to June 2021. KIRIS encompasses all inoculation data for 18 types of vaccines covered under the National Immunization Program (NIP). The NHID includes demographic and clinical information of the entire population of 50 million South Koreans.^14^ Using anonymized identifiers, we extracted variables including age at vaccination, date of vaccination, type of vaccine, dosing schedule information, route of vaccination, and site of vaccination from the KIRIS. Additionally, we obtained data on medical diagnosis, date of diagnosis, prescriptions, procedures, health care utilization records, medical insurance type, region of residence, and income level from the NHID. A detailed description of this large linked database is provided elsewhere.^15^

By linking these 2 data sources, we could leverage information on exposure and outcomes to assess the association between PPSV23 and adverse events. All procedures and prescriptions were coded using the national codes. Diagnostic information was coded according to the *International Statistical Classification of Diseases and Related Health Problems, Tenth Revision (ICD-10)*. A previously conducted validation study reported a positive predictive value of 82% for diagnosis codes in claims data compared with diagnoses from electronic medical records, which are regarded as the reference standard.^16^

### Study Population

Older adults aged 65 years or older who received PPSV23 between July 2018 and June 2021 were eligible for the study. Among these eligible individuals, we excluded individuals with the following conditions: those with a record that was inconsistent with current PPSV23 vaccination guidelines or NIP criteria for older adults (such as having received 2 or more doses of PPSV23, being younger than 65 years of age, or having pneumococcal conjugate vaccination records), missing information on age or sex, and those who died between the start date of the predefined risk interval and the end date of control intervals to ensure an adequate follow-up period for outcome assessment.

In South Korea, health care clinicians are required to report detailed information on immunizations to KIRIS to request reimbursement of immunization fees from the governmental body for all vaccines covered under the NIP. Therefore, records of PPSV23 immunization were obtained from KIRIS. Cohort entry was defined as the date of PPSV23 immunization. Baseline characteristics, such as age at vaccination, sex, region of residence, type of health insurance, income level, comorbidities, history of medication use, Charlson Comorbidity Index, health care utilization, and history of seasonal influenza vaccination were assessed at cohort entry.

### Outcomes

We defined 6 cardiovascular outcomes (myocardial infarction, atrial fibrillation, cardiomyopathy, heart failure, hypotension, myocarditis or pericarditis, and stroke), 2 neurological outcomes (Bell palsy and Guillain-Barré syndrome), and 3 immunological outcomes (sepsis, thrombocytopenia, and anaphylaxis) as the primary outcomes of interest. The selection of these outcomes was based on previous case series, postauthorization safety control studies, observational studies, and consensus from the Korean advisory committee on vaccine safety.^8,9,11,12,13,17^ Each outcome was identified using *ICD-10* diagnostic codes in either the primary or secondary position in the inpatient or emergency department settings. The detailed diagnostic codes are provided in eTable 1 in Supplement 1. We also applied additional criteria to ensure the validity of the outcome definitions. For thrombocytopenia, we excluded cases with a concurrent diagnosis of cancer recorded on the same day to distinguish chemotherapy-induced thrombocytopenia. To reduce false positives in anaphylaxis cases, we only included cases with a diagnosis code for anaphylaxis and simultaneous prescription of epinephrine or systemic steroids.^18^ Only incident cases were included in this study, and individuals with a history of each outcome diagnosis within 1 year before cohort entry were excluded.

### Self-Controlled Risk Interval Analysis

**Figure 1.**
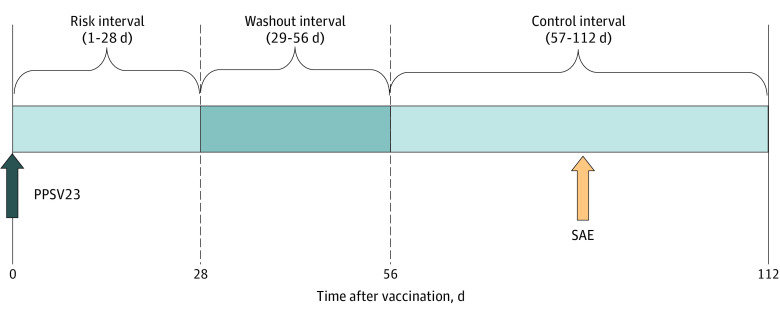
Study Design of Self-Controlled Risk Interval Analysis For sepsis and anaphylaxis, the risk intervals were defined as days 0 to 7 and 0 to 2, respectively, including the day of vaccination. PPSV23 indicates the 23-valent pneumococcal polysaccharide vaccine; SAE, serious adverse event.

We used a self-controlled risk interval (SCRI) design that included older adults with records of both PPSV23 vaccination and at least 1 of the predefined outcomes (Figure 1). This design allowed us to minimize the impact of time-invariant confounders and exposure misclassification by comparing the risk between specific time periods within individuals who experienced an outcome, rather than between different individuals. Moreover, it is less susceptible to the effects of unmeasured confounders than other study designs.^19^ Given the challenges in selecting appropriate comparator groups due to the heterogeneous characteristics of vaccinated and unvaccinated groups in older adults, the SCRI design can complement the limitations of cohort designs and serve as an alternative approach to assess the association of adverse events following PPSV23 vaccination.

The risk interval was defined as days 1 to 28 after PPSV23 vaccination, while the control interval was defined as days 57 to 112 after PPSV23 vaccination, with day 0 defined as the day of PPSV23 vaccination. The adoption of a 28-day risk period aligns with previous studies investigating the cardiovascular, neurological, or immunological events associated with vaccination.^20,21,22,23,24^ We excluded outcomes that occurred on the day of vaccination to account for the possibility of reverse causation, unless stated otherwise. Additionally, we implemented a 28-day washout interval between the risk and control intervals to address the possibility that adverse events occurring beyond the predefined risk interval might be influenced by prior PPSV23 vaccination. This measure is implemented to mitigate the underestimation of risk due to outcome misclassification.^3,19^ For sepsis and anaphylaxis, the risk intervals were defined as days 0 to 7 and 0 to 2, respectively, including the day of vaccination, based on the pathophysiology associated with these outcomes. The same washout and control intervals as those for other events were applied to enhance statistical power for these 2 events.

### Statistical Analyses

We summarized the sociodemographic, clinical, and vaccination-related characteristics of the study population. Categorical variables are presented as frequencies and proportions, while continuous variables are presented as means and SDs. To estimate the incidence rate ratio (IRR) with a 95% CI for each outcome, we used a conditional Poisson regression model. The calculation of IRR allowed us to adjust for differences in the durations between the risk and control intervals. The model was adjusted for age, which is a time-varying variable, throughout the risk and control intervals. Additionally, we presented the temporal distributions of case onset according to the number of days after PPSV23 vaccination to identify the overall clusters of adverse events following vaccination. All statistical analyses were performed using SAS Enterprise Guide 7.1 for Windows (SAS Institute). A 2-tailed *P* value of .05 was considered statistically significant. Data were analyzed from November 2022 to April 2023.

We conducted sensitivity analyses with different risk intervals (21, 42, 84, and 112 days) to ensure the robustness of our main analysis and to explore the possibility of capturing insidious outcomes that may not have been identified with the 28-day risk interval. We also varied the washout interval from 28 days to 14 or 42 days. To account for the potential impact of influenza vaccination or seasonality, we further adjusted for influenza vaccination status and seasonality during the risk and control intervals. Additionally, we conducted an analysis that included day 0 in the risk interval. Subgroup analyses were performed to assess the heterogeneity of the outcomes of PPSV23 vaccination based on patient characteristics such as sex, history of cardiovascular diseases (coronary artery disease, myocardial infarction, arrhythmia, hypertension, stroke, atherosclerosis, heart failure, and peripheral arterial disease), and immunocompromising status. These subgroup analyses allowed us to examine whether the association between PPSV23 vaccination and adverse events differed across specific subsets of populations.

## Results

**Figure 2.**
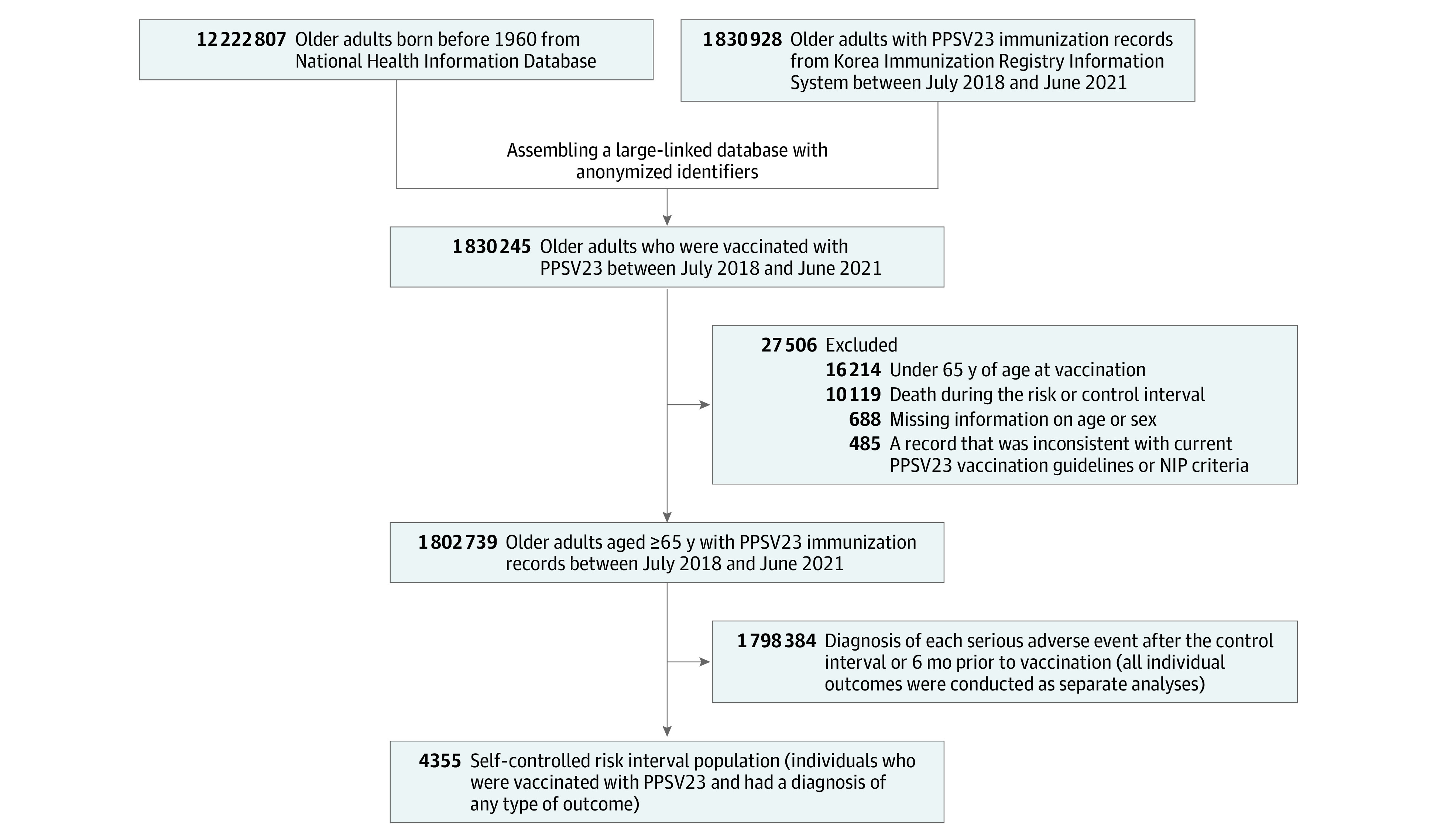
Flowchart of Patient Selection Criteria for Self-Controlled Risk Interval Analysis in a Large Linked Database PPSV23 indicates the 23-valent pneumococcal polysaccharide vaccine.

After linking the NHID of 12 222 807 individuals with the PPSV23 immunization records of 1 830 928 individuals from the KIRIS, the data of 1 830 245 adults were available (Figure 2). Of these, 27 506 were excluded for various reasons. Among the 1 802 739 individuals who received PPSV23, those who experienced outcome events of interest within 6 months before vaccination were excluded, and 4355 patients who experienced events during the risk or control intervals were included in the SCRI analysis.

Differential characteristics were observed between the SCRI population and PPSV23 vaccinated population. The mean (SD) age of the SCRI population was 72.4 (8.2) years, which was higher than that of the PPSV23 vaccinated population (68.3 [5.6] years) (Table). A total of 2272 patients were male (52.1%). The proportion of patients with comorbidities was higher in patients with events compared with the PPSV23 vaccinated population (3224 patients [74.0%], 548 patients [12.5%], and 1570 patients [36.0%] for cardiovascular disease, chronic lung disease, and diabetes, respectively). The proportion of patients who used systemic corticosteroids was 29.4% (1282 patients) and those using other immunosuppressants was 1.2% (50 patients). The mean (SD) Charlson Comorbidity Index was 1.66 (1.78), while that of PPSV23 vaccinated population was 1.01 (1.43). The SCRI population used more health care services than the PPSV23 vaccinated population. The proportion of medical aid in the SCRI population was almost double that in PPSV23 vaccinated population (11.0% [481 patients] vs 5.2% [92 752 patients]).

**Table.** Characteristics of Those Vaccinated With 23-Valent Pneumococcal Polysaccharide Vaccine (PPSV23) and Those Included in the Self-Controlled Risk Interval Analysis

Characteristics	Patients, No. (%)	
	Population vaccinated with PPSV23 (n = 1 802 739)	SCRI population (n = 4355)^a^
Age at vaccination, mean (SD)	68.26 (5.57)	72.44 (8.22)
Age group, y		
65-74	1 565 095 (86.8)	2799 (64.2)
75-84	192 424 (10.6)	1085 (24.9)
≥85	45 220 (2.5)	471 (10.8)
Sex		
Male	826 825 (45.8)	2272 (52.1)
Female	975 914 (54.1)	2083 (47.8)
Comorbidities		
Cardiovascular disease	1 090 880 (60.5)	3224 (74.0)
Chronic lung disease	148 714 (8.3)	548 (12.5)
Diabetes	509 482 (28.2)	1570 (36.0)
Chronic liver disease	204 480 (11.3)	505 (11.6)
Chronic kidney disease	85 113 (4.7)	482 (11.0)
Immunocompromised	181 223 (10.0)	506 (11.6)
CSF leak	49 (<1)	0
Cochlear implant	9 (<1)	0
Alcohol dependence	6677 (0.4)	32 (0.7)
Tobacco dependence	653 (0.04)	1 (0.02)
Pneumonia	41 927 (2.3)	259 (5.9)
Medication use		
Systemic corticosteroids	458 474 (25.4)	1282 (29.4)
Other immunosuppressants	12 811 (0.7)	50 (1.2)
Charlson Comorbidity Index, mean (SD)	1.01 (1.4)	1.66 (1.8)
Health care utilization, mean (SD)		
No. of outpatient visits	13.37 (14.4)	16.39 (19.4)
No. of hospital admissions	0.29 (1.1)	1.31 (2.7)
No. of ED visits	0.04 (0.3)	0.12 (0.5)
History of seasonal influenza vaccination	75 798 (4.2)	201 (4.6)
Region of residence		
Metropolitan^b^	880 556 (48.8)	1905 (43.7)
Provincial or rural	922 183 (51.1)	2450 (56.2)
Types of health insurance		
Local health insurance	565 762 (31.3)	1372 (31.5)
Employee health insurance	1 144 225 (63.4)	2502 (57.4)
Medical aid	92 752 (5.2)	481 (11.0)
Income level		
First quartile (most deprived)	408 400 (22.6)	1181 (27.1)
Second quartile	300 949 (16.6)	633 (14.5)
Third quartile	376 417 (20.8)	862 (19.7)
Fourth quartile (most affluent)	708 001 (39.2)	1656 (38.0)

Abbreviations: CSF, cerebrospinal fluid; ED, emergency department; SCRI, self-controlled risk interval.

^a^ Individuals who received PPSV23 and experienced at least 1 outcome of interest.

^b^ Ten large cities with a population of over 1 million.

**Figure 3.**
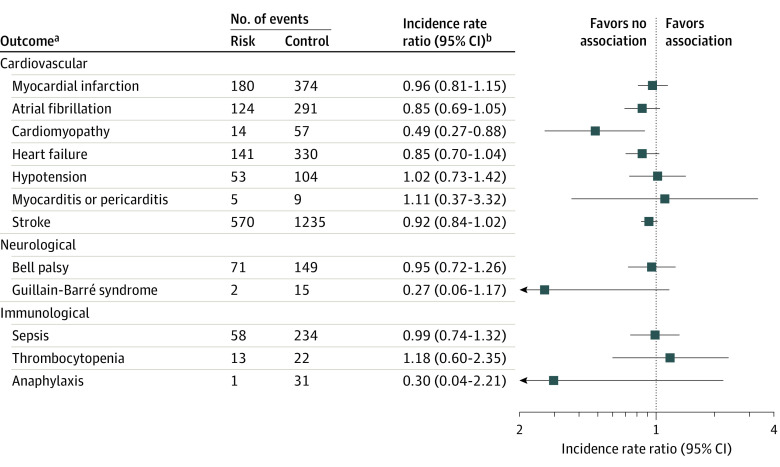
Self-Controlled Risk Interval Analysis of Cardiovascular, Neurological, and Immunological Outcomes Following 23-Valent Pneumococcal Polysaccharide Vaccination ^a^A risk interval of 28 days was applied unless otherwise specified in the main analysis. A washout interval of 28 days was applied between the risk and control intervals. For sepsis and anaphylaxis, risk intervals of 7 days and 2 days were defined, respectively, including the day of vaccination. ^b^Incidence rate ratio was adjusted for age.

In each SCRI analysis, we did not find an increased risk of cardiovascular, neurological, or immunological events following PPSV23 vaccination (Figure 3). Specifically, for cardiovascular events, null results were shown for myocardial infarction (IRR, 0.96; 95% CI, 0.81-1.15), atrial fibrillation (IRR, 0.85; 95% CI, 0.69-1.05), heart failure (IRR, 0.85; 95% CI, 0.70-1.04), hypotension (IRR, 1.02; 95% CI, 0.73-1.42), myocarditis or pericarditis (IRR, 1.11; 95% CI, 0.37-3.32), and stroke (IRR, 0.92; 95% CI, 0.84-1.02), while the risk of cardiomyopathy was significantly lower in risk interval (IRR, 0.49; 95% CI, 0.27-0.88). As for neurological events, Bell palsy (IRR, 0.95; 95% CI, 0.72-1.32) and Guillain-Barre syndrome (IRR, 0.27; 95% CI, 0.06-1.17) were not associated with PPSV23 vaccination. In terms of immunological events, PPSV23 did not increase the risk of sepsis (IRR, 0.99; 95% CI, 0.74-1.32), thrombocytopenia (IRR, 1.18; 95% CI, 0.60-2.35), or anaphylaxis (IRR, 0.30; 95% CI, 0.04-2.21).

**Figure 4.**
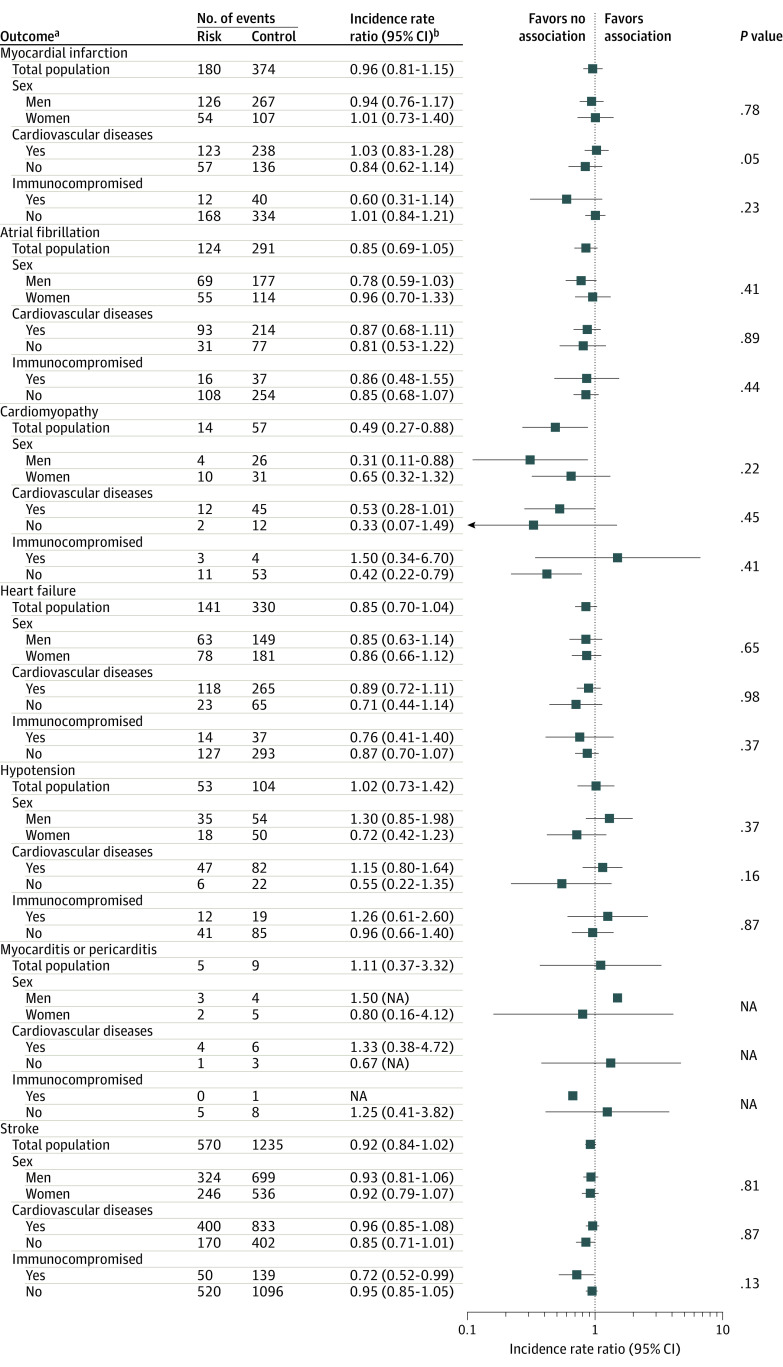
Subgroup Analysis of Cardiovascular Outcomes Following 23-Valent Pneumococcal Polysaccharide Vaccination NA indicates not available (model did not converge due to an insufficient number of outcomes). ^a^A risk interval of 28 days was applied. A washout interval of 28 days was applied between the risk and control intervals. ^b^Incidence rate ratio was adjusted for age.

None of the subgroups showed a significant positive association between PPSV23 and cardiovascular (Figure 4), neurological (eFigure 1 in Supplement 1), and immunological (eFigure 2 in Supplement 1) events. Although the risk of cardiovascular events tended to increase in patients with a history of cardiovascular diseases, the association was not statistically significant. The risk of immunological events was higher in immunocompromised patients, but the small number of events led to wide CIs (sepsis IRR, 1.38; 95% CI, 0.65-2.95; thrombocytopenia IRR, 2.00; 95% CI, 0.28-14.20; and anaphylaxis IRR, 2.33; 95% CI, 0.26-20.88). Results from the sensitivity analysis, including variations in risk or control or washout intervals, adjustment for influenza vaccination status or seasonality, and day 0 inclusion in the risk interval, were consistent with the main findings (eTable 2, eTable 3, and eTable 4 in Supplement 1). The case distribution of events according to the days after vaccination with PPSV23 did not show any temporal trends of increasing risk, as shown in eFigure 3, eFigure 4, and eFigure 5 in Supplement 1.

## Discussion

Our study found no notable association between vaccination with PPSV23 and the risk of most cardiovascular (myocardial infarction IRR, 0.96; 95% CI, 0.81-1.15; heart failure IRR, 0.85; 95% CI, 0.70-1.04; stroke IRR, 0.92; 95% CI, 0.84-1.02), neurological (Bell palsy IRR, 0.95; 95% CI, 0.72-1.26), or immunological (sepsis IRR, 0.99; 95% CI, 0.74-1.32; thrombocytopenia IRR, 1.18; 95% CI, 0.60-2.35) events. Even in patients with a history of cardiovascular diseases, vaccination did not lead to an increased risk of cardiovascular events. PPSV23 vaccination was likely associated with a higher risk of immunological adverse events such as sepsis, thrombocytopenia, and anaphylaxis in immunocompromised patients; however, the overall incidence of these events was very low. The results of the sensitivity analyses support the robustness of our main findings.

Many studies and accumulated clinical experience have supported the fact that vaccination with PPSV23 is safe, and our findings reinforce this evidence. Clinical trials conducted with older adults have not reported any serious adverse reactions related to PPSV23.^25,26,27^ Postlicensure studies using data from the Vaccine Adverse Event Reporting System in the US^13^ and General Practice data in Australia^28^ also did not find any unexpected severe systemic adverse events following PPSV23 vaccination. Our results, applying a self-controlled design, provide additional evidence that there was no increased risk of adverse events of interest after vaccination, even after adjusting for all time-invariant, unmeasured confounding factors by performing a within-person comparison.^29^

A previous Korean study using the Adverse Event Reporting System database identified unexpected safety signals for PPSV23, including cardiovascular and immunological events.^11^ In line with pharmacovigilance efforts,^30^ we conducted an epidemiological study to evaluate these signals but find no significant positive association between PPSV23 and adverse events. The discrepancy with the previous study may be attributed to different study designs; a signal does not indicate a causal relationship, and the information in a signal is not conclusive because it may change over time with additional data.^31^ The variation in age distribution among study populations could also contribute to the observed inconsistency. The former study included younger adults (19-65 years), whereas our focus was on older adults. Guidelines recommend PPSV23 for younger adults with risk factors for pneumonia,^32^ potentially leading to a higher incidence of adverse events due to confounding by indication. Differences in the incidence of myocardial infarction and stroke between age groups after PPSV23 receipt were also identified in a US study.^8^ Future studies involving younger adults are required to assess the risk of serious adverse events associated with PPSV23 in different age groups.

In a US cohort study using the Vaccine Safety Datalink project data, safety outcomes including acute myocardial infarction, atrial fibrillation, cardiomyopathy, heart failure, Guillain-Barre syndrome, thrombocytopenia, and allergic reactions in the elderly were less favorable with PPSV23 compared with pneumococcal conjugate vaccine 13 (PCV13),^33^ but were comparable with the general US population aged 65 years or older.^34^ We also assessed the association between PPSV23 and outcomes using a nationwide linked database in Korea, demonstrating favorable overall safety. In our study, among the total PPSV23 vaccinated population, 180 (0.01%) experienced myocardial infarction within the risk interval (28 days after vaccination). Considering the incidence rates of myocardial infarction in the Korean population aged older than 60 years (1.18-3.21 per 1000 person-year),^35^ the occurrence in this study also seemed similar to that in the overall population.

### Limitations and Strengths

This study had several limitations. First, the risk of adverse events may have been underestimated because patients who received more than 1 dose of PPSV23 (ie, prevalent users) were included in this study. Our data only had NIP-supported vaccination records; thus, we could not exclude patients who paid out of pocket for PPSV23 (before age 65 years) or PCV13. These prevalent users can contribute to mitigating early harm following drug exposure as survivors of the initial treatment.^36^ Second, the self-controlled design, involving only vaccinees, might also underestimate risk due to potential selection bias from including individuals eligible for vaccination. We also included individuals alive during the risk and control intervals, implying that vaccinees who died after adverse events in the risk period were excluded. Consequently, the risk of adverse events in the risk interval might be lower than that in the control interval among this population. However, we tried to reduce healthy vaccinee bias by excluding time period immediately before vaccination from our analysis.^19^ Third, the adverse events caused by the administration of PPSV23 may have occurred after the risk interval. We attempted to set the plausible lengths of the risk, washout, and control intervals by considering physiological mechanisms, but these operational definitions may have led to outcome misclassification. Nonetheless, this bias was unlikely to have substantially impacted our results, given the consistent results from the sensitivity analysis with varying interval lengths.

Despite limitations, our study has generalizability, using a nationwide large linked database with over 1.8 million PPSV23 recipients. To our knowledge, this is the first self-controlled risk interval study to investigate the safety of PPSV23. The risk interval design, focusing solely on vaccinated individuals, reduces bias from unmeasured differences that may exist between vaccinees and the unvaccinated, a limitation of the cohort design. Our findings align with those of previous cohort studies, supporting the overall safety of PPSV23 across diverse outcomes and study designs.

## Conclusions

In this self-controlled study, our results corroborate previous findings that PPSV23 was not associated with a higher risk of serious systemic adverse events related to the cardiovascular, neurological, and immunological systems. Even among adults with a history of cardiovascular diseases or immunocompromised status, PPSV23 did not appear to elevate the risk of related events to worrying levels. These updated safety findings on PPSV23 can help alleviate concerns regarding the vaccine and encourage vaccination in elderly individuals.

## Notes

Supplement 1. **eTable 1. **Definitions of Outcomes and Comorbidities in the Study **eTable 2. **Results of Sensitivity Analysis Varying Risk Period **eTable 3. **Results of Sensitivity Analysis Varying Washout Period **eTable 4. **Results of Sensitivity Analysis Varying Other Settings **eFigure 1. **Subgroup Analysis of Neurological Outcomes Following 23-Valent Pneumococcal Polysaccharide Vaccination **eFigure 2. **Subgroup Analysis of Immunological Outcomes Following 23-Valent Pneumococcal Polysaccharide Vaccination **eFigure 3. **Case Distributions of Cardiovascular Outcomes According to Days After Vaccination of the 23-Valent Pneumococcal Polysaccharide Vaccine **eFigure 4. **Case Distributions of Neurological Outcomes According to Days After Vaccination of the 23-Valent Pneumococcal Polysaccharide Vaccine **eFigure 5. **Case Distributions of Immunological Outcomes According to Days After Vaccination of the 23-Valent Pneumococcal Polysaccharide Vaccine

Supplement 2.
Data Sharing Statement
