# Supplementary material for: Cardiovascular, Neurological, and Immunological Adverse Events and the 23-Valent Pneumococcal Polysaccharide Vaccine
Source: JAMA Netw Open. 2024 Jan 22;7(1):e2352597. doi: 10.1001/jamanetworkopen.2023.52597 (PMC10804273; doi:10.1001/jamanetworkopen.2023.52597)
Supplement: Supplement 1. — eTable 1. Definitions of Outcomes and Comorbidities in the Study eTable 2. Results of Sensitivity Analysis Varying Risk Period eTable 3. Results of Sensitivity Analysis Varying Washout Period eTable 4. Results of Sensitivity Analysis Varying Other Settings eFigure 1. Subgroup Analysis of Neurological Outcomes Following 23-Valent Pneumococcal Polysaccharide Vaccination eFigure 2. Subgroup Analysis of Immunological Outcomes Following 23-Valent Pneumococcal Polysaccharide Vaccination eFigure 3. Case Distributions of Cardiovascular Outcomes According to Days After Vaccination of the 23-Valent Pneumococcal Polysaccharide Vaccine eFigure 4. Case Distributions of Neurological Outcomes According to Days After Vaccination of the 23-Valent Pneumococcal Polysaccharide Vaccine eFigure 5. Case Distributions of Immunological Outcomes According to Days After Vaccination of the 23-Valent Pneumococcal Polysaccharide Vaccine [file jamanetwopen-e2352597-s001.pdf]

Supplemental Online Content

Yoon D, Jeon H-L, Kim JH, Lee H, Shin J-Y. Cardiovascular, neurological, and immunological adverse events and the 23-valent pneumococcal polysaccharide vaccine. *JAMA Netw Open*. 2024;7(1):e2352597. doi:10.1001/jamanetworkopen.2023.52597

eTable 1. Definitions of Outcomes and Comorbidities in the Study
eTable 2. Results of Sensitivity Analysis Varying Risk Period
eTable 3. Results of Sensitivity Analysis Varying Washout Period
eTable 4. Results of Sensitivity Analysis Varying Other Settings
eFigure 1. Subgroup Analysis of Neurological Outcomes Following 23-Valent Pneumococcal Polysaccharide Vaccination
eFigure 2. Subgroup Analysis of Immunological Outcomes Following 23-Valent Pneumococcal Polysaccharide Vaccination
eFigure 3. Case Distributions of Cardiovascular Outcomes According to Days After Vaccination of the 23-Valent Pneumococcal Polysaccharide Vaccine
eFigure 4. Case Distributions of Neurological Outcomes According to Days After Vaccination of the 23-Valent Pneumococcal Polysaccharide Vaccine
eFigure 5. Case Distributions of Immunological Outcomes According to Days After Vaccination of the 23-Valent Pneumococcal Polysaccharide Vaccine

This supplemental material has been provided by the authors to give readers additional information about their work.

© 2024 Yoon D et al. *JAMA Network Open.*

eTable 1. Definitions of Outcomes and Comorbidities in the Study

Outcomes	ICD-10 codes
**Cardiovascular**	
Myocardial infarction	I21, I22
Atrial fibrillation	I48
Cardiomyopathy	I42, I43
Heart failure	I50
Hypotension	I95
Myocarditis or pericarditis	I40, I41, I514, I010, I092, I30, I310, I311, I32
Stroke	I60, I61, I62, I63, I64
**Neurological**	
Bell's palsy	G510
Guillain-Barré syndrome	G610
**Immunological**	
Sepsis	A41, A40, R572, R65
Thrombocytopenia	D691, D693, D694, D695, D696
Anaphylaxis	T782, T886
**Comorbidities**	
Cardiovascular disease	I20, I21, I22, I23, I24, I25, I110, I130, I132, I49, I50, I10, I11, I12, I13, I15, I60, I61, I62, I63, I64, I65, I66, I67, I68, I69, I70, I73
Chronic lung disease	J43-46
Diabetes	E10-E14
Chronic liver disease	K704, K704, K711, K713, K714, K715, K721, K743, K767, K768, K769, K73
Chronic kidney disease	E112, E132, E142, I12-I13, N00-N08, N17-N19, N25-N27
Immunocompromised	T86, Z94, C00-C80, C81, C82, C83, C84, C85, C86, C87, C88, C89, C90, C91, C92, C93, C94, C95, C96, B20, B21, B22, B23, B24, M05, M06, M082, M083, M084, M32, G35, K50, K51, L40, L40.5, M07.0, M07.1, M07.2, M09.0
CSF leak	G960
Cochlear implant	Z9620
Alcohol dependence	F10-F16, F18-F19, Z71.4, Z71.5, Z72.1, Z72.2
Tobacco dependence	F17, Z71.6, Z72.0
Pneumonia	J12-J18

© 2024 Yoon D et al. *JAMA Network Open.*

eTable 2. Results of Sensitivity Analysis Varying Risk Period

Outcome[a]	Events, No		Incidence rate ratio[b] (95% CI)
	Risk	Control	
**Cardiovascular**			
Myocardial infarction			
Main analysis	180	374	0.96 (0.81 - 1.15)
Risk period of 21 days	131	350	1.00 (0.82 - 1.22)
Risk period of 42 days	288	293	0.98 (0.84 - 1.16)
Risk period of 84 days	570	579	0.98 (0.88 - 1.11)
Risk period of 112 days	760	828	0.92 (0.83 - 1.01)
Atrial fibrillation			
Main analysis	124	291	0.85 (0.69 - 1.05)
Risk period of 21 days	96	305	0.84 (0.67 - 1.06)
Risk period of 42 days	209	232	0.90 (0.75 - 1.09)
Risk period of 84 days	432	473	0.91 (0.80 - 1.04)
Risk period of 112 days	580	678	0.86 (0.77 - 0.96)
Cardiomyopathy			
Main analysis	14	57	0.49 (0.27 - 0.88)
Risk period of 21 days	12	57	0.56 (0.30 - 1.05)
Risk period of 42 days	26	41	0.63 (0.39 - 1.04)
Risk period of 84 days	72	88	0.82 (0.60 - 1.12)
Risk period of 112 days	96	127	0.76 (0.58 - 0.99)
Heart failure			
Main analysis	141	330	0.85 (0.70 - 1.04)
Risk period of 21 days	102	322	0.84 (0.68 - 1.06)
Risk period of 42 days	229	256	0.89 (0.75 - 1.07)
Risk period of 84 days	479	572	0.84 (0.74 - 0.95)
Risk period of 112 days	646	758	0.85 (0.77 - 0.95)
Hypotension			
Main analysis	53	104	1.02 (0.73 - 1.42)
Risk period of 21 days	38	98	1.03 (0.71 - 1.50)
Risk period of 42 days	82	84	0.98 (0.72 - 1.32)
Risk period of 84 days	147	128	1.15 (0.91 - 1.46)
Risk period of 112 days	209	197	1.06 (0.87 - 1.29)
Myocarditis or pericarditis			
Main analysis	5	9	1.11 (0.37 - 3.32)
Risk period of 21 days	2	7	0.76 (0.16 - 3.67)
Risk period of 42 days	5	7	0.71 (0.23 - 2.25)
Risk period of 84 days	8	12	0.67 (0.27 - 1.63)
Risk period of 112 days	15	17	0.88 (0.44 - 1.77)
Stroke			

© 2024 Yoon D et al. *JAMA Network Open.*

Main analysis	570	1235	0.92 (0.84 - 1.02)
Risk period of 21 days	423	1236	0.91 (0.82 - 1.02)
Risk period of 42 days	884	902	0.98 (0.89 - 1.08)
Risk period of 84 days	1817	1886	0.96 (0.90 - 1.03)
Risk period of 112 days	2419	2577	0.94 (0.89 - 0.99)
**Neurological**			
Bell's palsy			
Main analysis	71	149	0.95 (0.72 - 1.26)
Risk period of 21 days	52	154	0.90 (0.66 - 1.23)
Risk period of 42 days	97	124	0.78 (0.60 - 1.02)
Risk period of 84 days	197	195	1.01 (0.83 - 1.23)
Risk period of 112 days	278	274	1.01 (0.86 - 1.20)
Guillain-Barré syndrome			
Main analysis	2	15	0.27 (0.06 - 1.17)
Risk period of 21 days	2	18	0.30 (0.07 - 1.28)
Risk period of 42 days	3	8	0.38 (0.10 - 1.41)
Risk period of 84 days	20	11	1.82 (0.87 - 3.79)
Risk period of 112 days	23	15	1.53 (0.80 - 2.94)
**Immunological**			
Sepsis			
Main analysis (Risk period of 7 days)	58	234	0.99 (0.74 - 1.32)
Risk period of 14 days	123	236	1.04 (0.84 - 1.30)
Risk period of 21 days	173	463	1.00 (0.84 - 1.19)
Risk period of 28 days	173	220	1.05 (0.86 - 1.28)
Risk period of 42 days	232	456	1.02 (0.87 - 1.19)
Thrombocytopenia			
Main analysis	13	22	1.18 (0.60 - 2.35)
Risk period of 21 days	12	25	1.28 (0.64 - 2.55)
Risk period of 42 days	23	13	1.77 (0.90 - 3.49)
Risk period of 84 days	43	38	1.13 (0.73 - 1.75)
Risk period of 112 days	50	56	0.89 (0.61 - 1.31)
Anaphylaxis			
Main analysis (Risk period of 2 days)	1	31	0.30 (0.04 - 2.21)
Risk period of 7 days	6	30	0.70 (0.29 - 1.68)
Risk period of 14 days	17	39	0.81 (0.46 - 1.44)
Risk period of 21 days	23	94	0.62 (0.39 - 0.98)

Abbreviations: No, number; CI, confidence interval;

[a]A risk interval of 28 days was applied unless otherwise specified in the main analysis. A washout interval of 28 days was applied between the risk and control intervals. For sepsis and anaphylaxis, risk intervals of 7 days and 2 days were defined, respectively, including the day of vaccination.

[b]Incidence rate ratio was adjusted for age.

© 2024 Yoon D et al. *JAMA Network Open.*

eTable 3. Results of Sensitivity Analysis Varying Washout Period

**Outcome**[a]	**Events, No**		**Incidence rate ratio**[b] **(95% CI)**
	**Risk**	**Control**	
**Cardiovascular**			
Myocardial infarction			
Main analysis	180	374	0.96 (0.81 - 1.15)
Wash-out period of 14 days	180	372	0.97 (0.81 - 1.16)
Wash-out period of 42 days	180	405	0.89 (0.75 - 1.06)
Atrial fibrillation			
Main analysis	124	291	0.85 (0.69 - 1.05)
Wash-out period of 14 days	124	311	0.80 (0.65 - 0.98)
Wash-out period of 42 days	124	296	0.84 (0.68 - 1.03)
Cardiomyopathy			
Main analysis	14	57	0.49 (0.27 - 0.88)
Wash-out period of 14 days	14	59	0.47 (0.27 - 0.85)
Wash-out period of 42 days	14	62	0.45 (0.25 - 0.81)
Heart failure			
Main analysis	141	330	0.85 (0.70 - 1.04)
Wash-out period of 14 days	141	327	0.86 (0.71 - 1.05)
Wash-out period of 42 days	141	353	0.80 (0.66 - 0.97)
Hypotension			
Main analysis	53	104	1.02 (0.73 - 1.42)
Wash-out period of 14 days	53	89	1.19 (0.85 - 1.67)
Wash-out period of 42 days	53	101	1.05 (0.75 - 1.46)
Myocarditis or pericarditis			
Main analysis	5	9	1.11 (0.37 - 3.32)
Wash-out period of 14 days	5	4	2.50 (N/A)
Wash-out period of 42 days	5	10	1.00 (0.34 - 2.93)
Stroke			
Main analysis	570	1235	0.92 (0.84 - 1.02)
Wash-out period of 14 days	570	1242	0.92 (0.83 - 1.01)
Wash-out period of 42 days	570	1217	0.94 (0.85 - 1.03)
**Neurological**			
Bell's palsy			
Main analysis	71	149	0.95 (0.72 - 1.26)
Wash-out period of 14 days	71	142	1.00 (0.75 - 1.33)
Wash-out period of 42 days	71	157	0.90 (0.68 - 1.20)
Guillain-Barré syndrome			
Main analysis	2	15	0.27 (0.06 - 1.17)
Wash-out period of 14 days	2	20	0.20 (0.05 - 0.86)
Wash-out period of 42 days	2	8	0.50 (0.11 - 2.35)
**Immunological**			
Sepsis			
Main analysis	58	234	0.99 (0.74 - 1.32)

© 2024 Yoon D et al. *JAMA Network Open.*

Wash-out period of 14 days	58	239	0.97 (0.73 - 1.29)
Wash-out period of 42 days	58	220	1.05 (0.79 - 1.41)
Thrombocytopenia			
Main analysis	13	22	1.18 (0.60 - 2.35)
Wash-out period of 14 days	13	23	1.13 (0.57 - 2.23)
Wash-out period of 42 days	13	25	1.04 (0.53 - 2.03)
Anaphylaxis			
Main analysis	1	31	0.30 (0.04 - 2.21)
Wash-out period of 14 days	1	37	0.25 (0.03 - 1.84)
Wash-out period of 42 days	1	40	0.23 (0.03 - 1.70)

Abbreviations: No, number; CI, confidence interval; N/A, not available (model did not converge due to insufficient number of outcomes)

[a]A risk interval of 28 days was applied unless otherwise specified in the main analysis. A washout interval of 28 days was applied between the risk and control intervals. For sepsis and anaphylaxis, risk intervals of 7 days and 2 days were defined, respectively, including the day of vaccination.

[b]Incidence rate ratio was adjusted for age.

eTable 4. Results of Sensitivity Analysis Varying Other Settings

Outcome[a]	Events, No		Incidence rate ratio[b] (95% CI)
	Risk	Control	
**Cardiovascular**			
Myocardial infarction			
Main analysis	180	374	0.96 (0.81 - 1.15)
Adjusted for influenza vaccination status	180	374	0.96 (0.80 - 1.14)
Adjusted for seasonality	180	374	0.97 (0.81 - 1.15)
Including outcome occurring on the Day 0	181	366	0.94 (0.78 - 1.12)
Atrial fibrillation			
Main analysis	124	291	0.85 (0.69 - 1.05)
Adjusted for influenza vaccination status	124	291	0.85 (0.69 - 1.05)
Adjusted for seasonality	124	291	0.89 (0.72 - 1.10)
Including outcome occurring on the Day 0	125	283	0.84 (0.68 - 1.03)
Cardiomyopathy			
Main analysis	14	57	0.49 (0.27 - 0.88)
Adjusted for influenza vaccination status	14	57	0.49 (0.27 - 0.87)
Adjusted for seasonality	14	57	0.52 (0.29 - 0.94)
Including outcome occurring on the Day 0	14	54	0.49 (0.27 - 0.89)
Heart failure			
Main analysis	141	330	0.85 (0.70 - 1.04)
Adjusted for influenza vaccination status	141	330	0.84 (0.69 - 1.02)
Adjusted for seasonality	141	330	0.84 (0.69 - 1.03)
Including outcome occurring on the Day 0	141	318	0.84 (0.69 - 1.03)
Hypotension			
Main analysis	53	104	1.02 (0.73 - 1.42)
Adjusted for influenza vaccination status	53	104	0.98 (0.71 - 1.36)
Adjusted for seasonality	53	104	0.97 (0.70 - 1.34)
Including outcome occurring on the Day 0	54	101	1.01 (0.73 - 1.41)
Myocarditis or pericarditis			
Main analysis	5	9	1.11 (0.37 - 3.32)
Adjusted for influenza vaccination status	5	9	1.03 (N/A)
Adjusted for seasonality	5	9	1.08 (0.43 - 2.69)
Including outcome occurring on the Day 0	5	8	1.19 (0.39 - 3.62)
Stroke			
Main analysis	570	1235	0.92 (0.84 - 1.02)
Adjusted for influenza vaccination status	570	1235	0.91 (0.82 - 1.01)
Adjusted for seasonality	570	1235	0.93 (0.84 - 1.03)
Including outcome occurring on the Day 0	582	1209	0.91 (0.83 - 1.01)
**Neurological**			
Bell's palsy			
Main analysis	71	149	0.95 (0.72 - 1.26)
Adjusted for influenza vaccination status	71	149	0.93 (0.70 - 1.23)
Adjusted for seasonality	71	149	0.92 (0.69 - 1.22)
Including outcome occurring on the Day 0	71	149	0.90 (0.68 - 1.20)
Guillain-Barré syndrome			
Main analysis	2	15	0.27 (0.06 - 1.17)
Adjusted for influenza vaccination status	2	15	0.25 (N/A)
Adjusted for seasonality	2	15	0.27 (0.06 - 1.18)

© 2024 Yoon D et al. *JAMA Network Open.*

Including outcome occurring on the Day 0	2	15	0.25 (0.06 - 1.11)
**Immunological**			
Sepsis			
Main analysis	58	234	0.99 (0.74 - 1.32)
Adjusted for influenza vaccination status	58	234	0.99 (0.74 - 1.32)
Adjusted for seasonality	58	234	1.00 (0.75 - 1.33)
Thrombocytopenia			
Main analysis	13	22	1.18 (0.60 - 2.35)
Adjusted for influenza vaccination status	13	22	1.18 (0.60 - 2.32)
Adjusted for seasonality	13	22	1.44 (0.72 - 2.88)
Including outcome occurring on the Day 0	13	22	1.12 (0.56 - 2.22)
Anaphylaxis			
Main analysis	1	31	0.30 (0.04 - 2.21)
Adjusted for influenza vaccination status	1	31	0.30 (0.04 - 2.22)
Adjusted for seasonality	1	31	0.29 (0.04 - 2.16)

Abbreviations: No, number; CI, confidence interval; N/A, not available (model did not converge due to insufficient number of outcomes)

[a]A risk interval of 28 days was applied unless otherwise specified in the main analysis. A washout interval of 28 days was applied between the risk and control intervals. For sepsis and anaphylaxis, risk intervals of 7 days and 2 days were defined, respectively, including the day of vaccination.

[b]Incidence rate ratio was adjusted for age.

eFigure 1. Subgroup Analysis of Neurological Outcomes Following 23-Valent Pneumococcal Polysaccharide Vaccination

Outcome[a]	Events, No Risk	Events, No Control	Incidence rate ratio[b] (95% CI)	P-value
Bell's palsy				
Total population	71	149	0.95 (0.72 - 1.26)	
Sex				0.65
Men	32	71	0.90 (0.59 - 1.37)	
Women	39	78	1.00 (0.68 - 1.47)	
Cardiovascular diseases				0.83
Yes	42	94	0.89 (0.62 - 1.29)	
No	29	55	1.05 (0.67 - 1.65)	
Immunocompromised				0.80
Yes	7	17	0.82 (0.34 - 1.99)	
No	64	132	0.97 (0.72 - 1.31)	
Guillain-Barré syndrome				
Total population	2	15	0.27 (0.06 - 1.17)	
Sex				0.32
Men	1	6	0.33 (0.04 - 2.77)	
Women	1	9	0.22 (0.03 - 1.75)	
Cardiovascular diseases				N/A
Yes	0	10	N/A	
No	2	5	0.80 (0.16 - 4.12)	
Immunocompromised				0.55
Yes	1	2	1.00 (0.09 - 11.03)	
No	1	13	0.15 (0.02 - 1.18)	

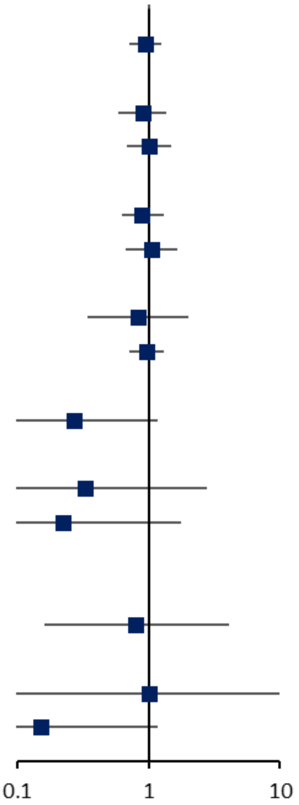

Abbreviations: CI, confidence interval; N/A, not available (model did not converge due to an insufficient number of outcomes).

[a]A risk interval of 28 days was applied. A washout interval of 28 days was applied between the risk and control intervals.

[b]Incidence rate ratio was adjusted for age.

© 2024 Yoon D et al. *JAMA Network Open.*

eFigure 2. Subgroup Analysis of Immunological Outcomes Following 23-Valent Pneumococcal Polysaccharide Vaccination

Outcome[a]	Events, No Risk	Events, No Control	Incidence rate ratio[b] (95% CI)	P-value
Sepsis				
Total population	58	234	0.99 (0.74 - 1.32)	
Sex				0.11
Men	27	100	1.08 (0.71 - 1.65)	
Women	31	134	0.93 (0.63 - 1.37)	
Cardiovascular diseases				0.23
Yes	44	181	0.97 (0.70 - 1.35)	
No	14	53	1.06 (0.59 - 1.90)	
Immunocompromised				0.08
Yes	9	26	1.38 (0.65 - 2.95)	
No	49	208	0.94 (0.69 - 1.29)	
Thrombocytopenia				
Total population	13	22	1.18 (0.60 - 2.35)	
Sex				0.57
Men	8	10	1.60 (0.63 - 4.05)	
Women	5	12	0.83 (0.29 - 2.37)	
Cardiovascular diseases				0.35
Yes	8	17	0.94 (0.41 - 2.18)	
No	5	5	2.00 (0.58 - 6.91)	
Immunocompromised				0.61
Yes	2	2	2.00 (0.28 - 14.20)	
No	11	20	1.10 (0.53 - 2.30)	
Anaphylaxis				
Total population	1	31	0.30 (0.04 - 2.21)	
Sex				N/A
Men	1	20	0.47 (0.06 - 3.48)	
Women	0	11	N/A	
Cardiovascular diseases				N/A
Yes	1	17	0.55 (0.07 - 4.13)	
No	0	14	N/A	
Immunocompromised				N/A
Yes	1	4	2.33 (0.26 - 20.88)	
No	0	27	N/A	

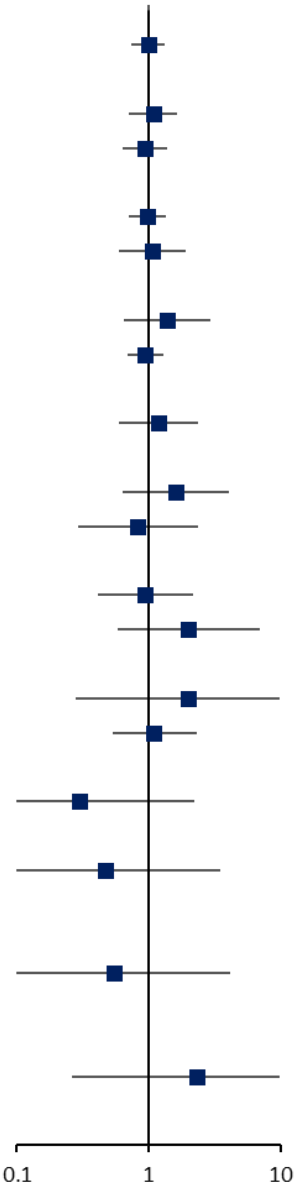

Abbreviations: CI, confidence interval; N/A, not available (model did not converge due to an insufficient number of outcomes).

[a]For sepsis and anaphylaxis, risk intervals of 7 days and 2 days were defined, respectively, including the day of vaccination. A risk interval of 28 days was applied for thrombocytopenia. A washout interval of 28 days was applied between the risk and control intervals.

[b]Incidence rate ratio was adjusted for age.

© 2024 Yoon D et al. *JAMA Network Open.*

eFigure 3. Case Distributions of Cardiovascular Outcomes According to Days After Vaccination of the 23-Valent Pneumococcal Polysaccharide Vaccine

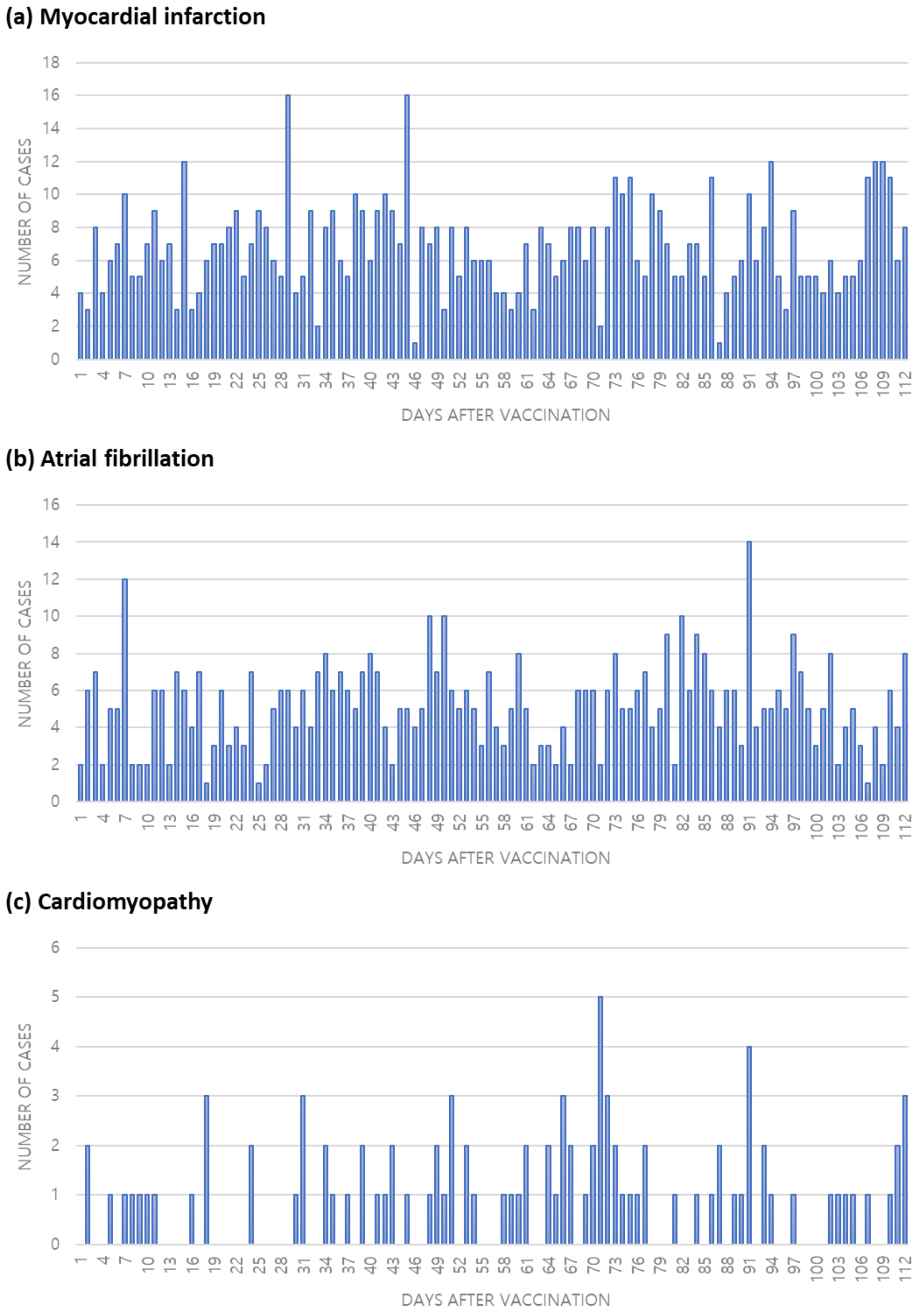

© 2024 Yoon D et al. *JAMA Network Open.*

(d) Heart failure

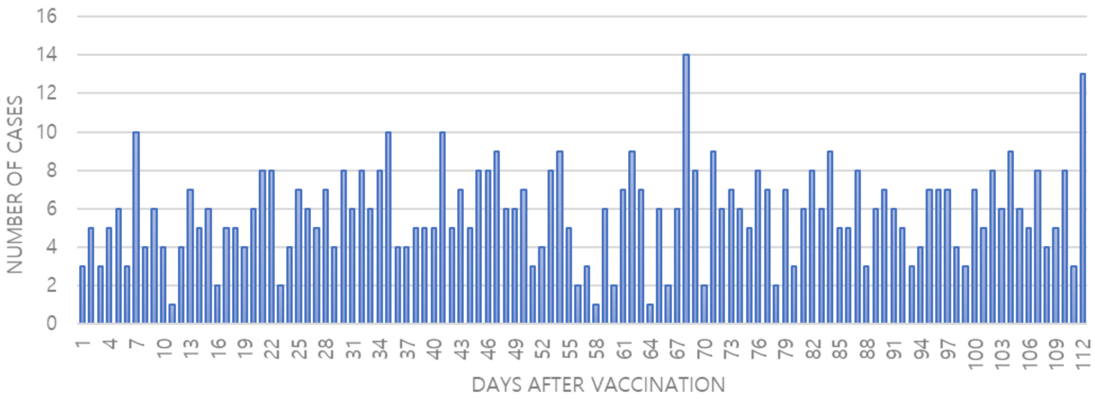

(e) Hypotension

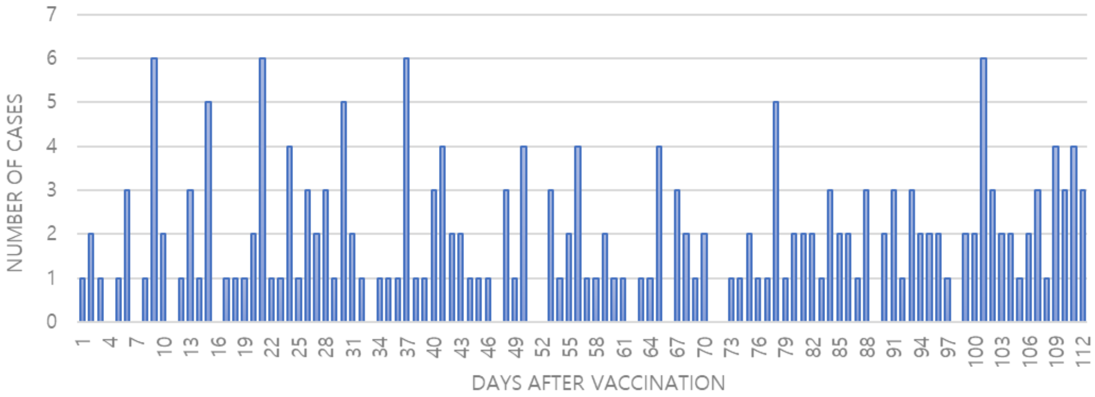

(f) Myocarditis or pericarditis

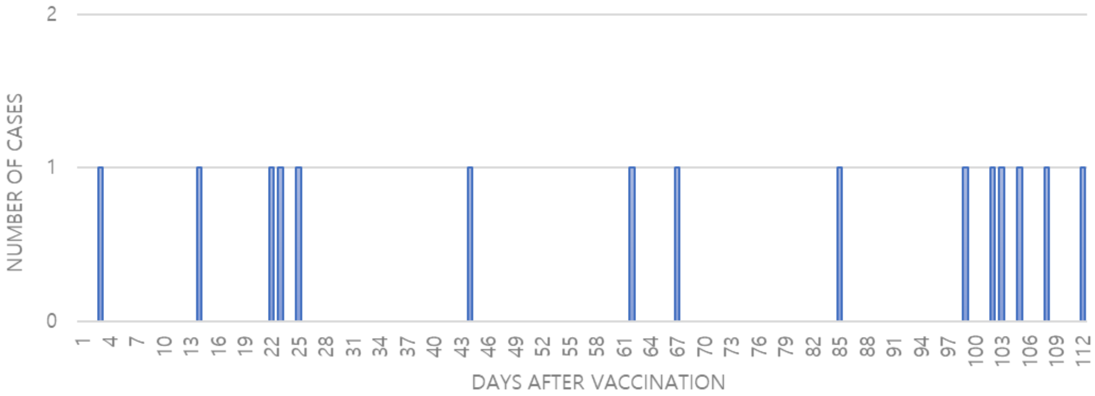

(g) Stroke

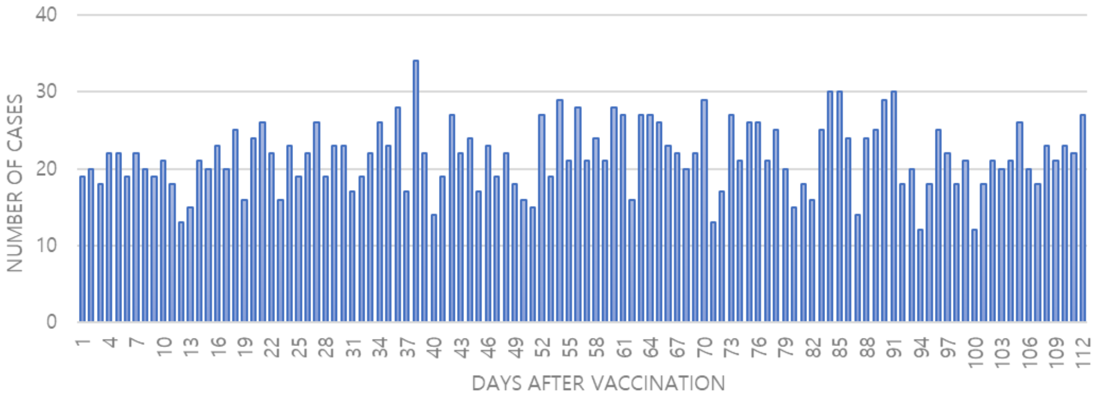

© 2024 Yoon D et al. *JAMA Network Open.*

eFigure 4. Case Distributions of Neurological Outcomes According to Days After Vaccination of the 23-Valent Pneumococcal Polysaccharide Vaccine

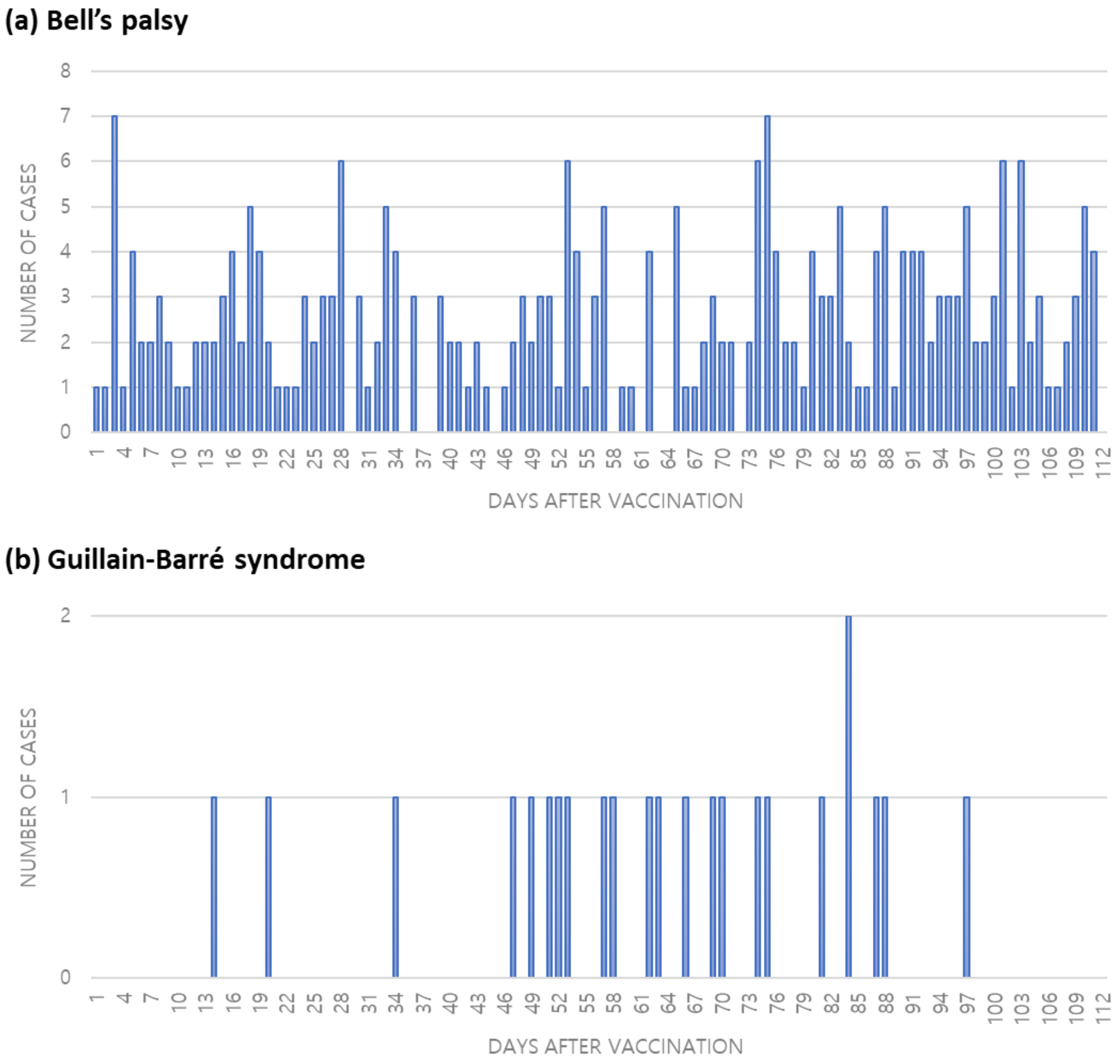

© 2024 Yoon D et al. *JAMA Network Open.*

eFigure 5. Case Distributions of Immunological Outcomes According to Days After Vaccination of the 23-Valent Pneumococcal Polysaccharide Vaccine

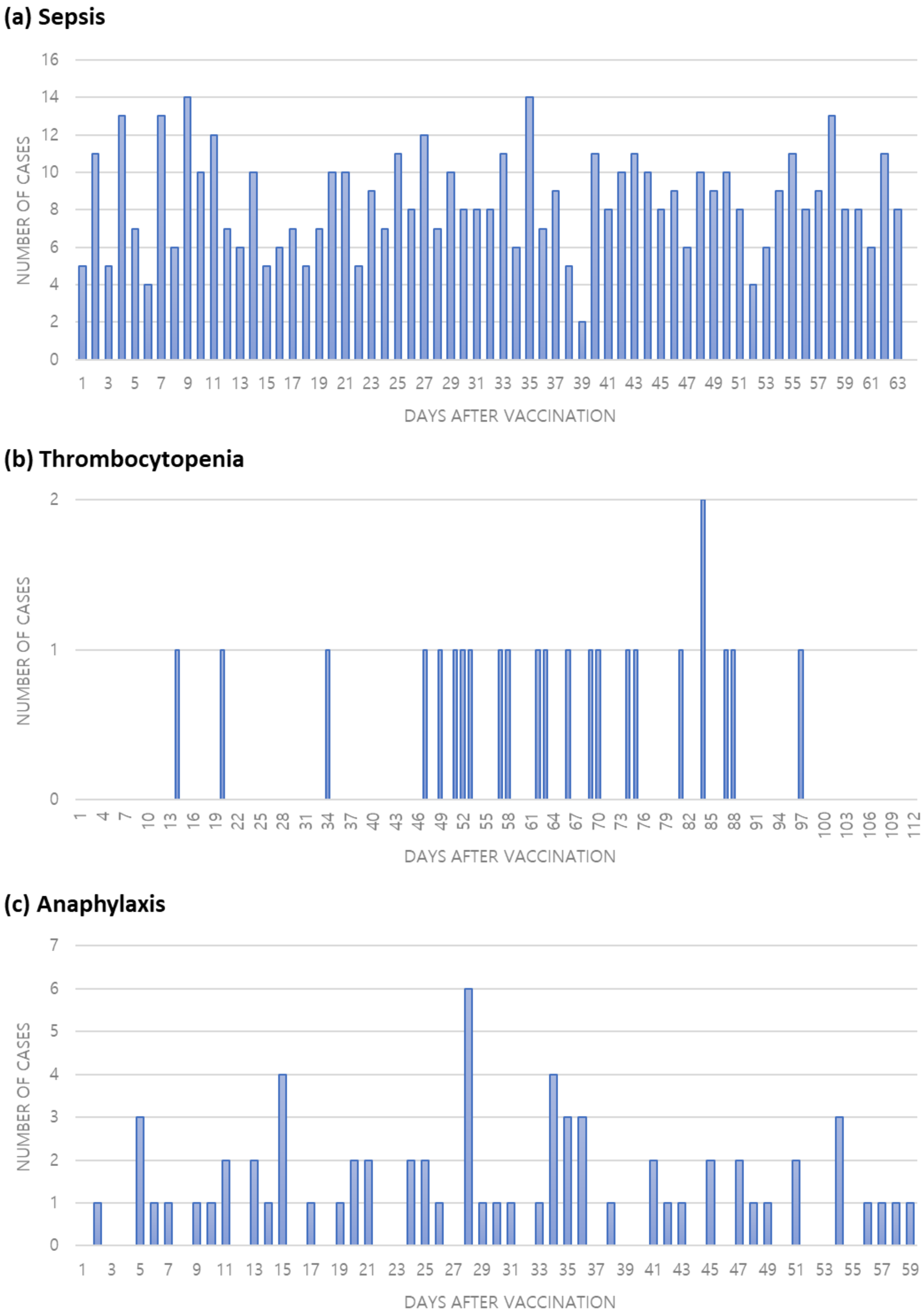

© 2024 Yoon D et al. *JAMA Network Open.*
