# Supplementary material for: Cardiovascular, Neurological, and Immunological Adverse Events and the 23-Valent Pneumococcal Polysaccharide Vaccine
Source: JAMA Netw Open. 2024 Jan 22;7(1):e2352597. doi: 10.1001/jamanetworkopen.2023.52597 (PMC10804273; doi:10.1001/jamanetworkopen.2023.52597)
Supplement: Supplement 2. — Data Sharing Statement [file jamanetwopen-e2352597-s002.pdf]

Data Sharing Statement

Yoon. Cardiovascular, Neurological, and Immunological Adverse Events and the 23-Valent Pneumococcal Polysaccharide Vaccine. *JAMA Netw Open*. Published January 22, 2024. doi:10.1001/jamanetworkopen.2023.52597

Data
Data available: No
